# Sheep-Associated Malignant Catarrhal Fever in an Exotic Rusa Deer (*Cervus timorensis*): Implications for Wildlife Health in Southern Brazil

**DOI:** 10.3390/microorganisms14102212

**Published:** 2026-10-01

**Authors:** Selwyn Arlington Headley, Maria Constanza Rodriguez, Fernanda Pinto-Ferreira, Alais Maria Dall Agnol, Flavia Helena Pereira Silva, Geovana Depieri Yoshitani, Amauri Alcindo Alfieri, Renato Silva de Sousa

**Affiliations:** 1 Laboratory of Animal Pathology, Department of Preventive Veterinary Medicine, Universidade Estadual de Londrina, Londrina 86057-970, Paraná, Brazil; flaviahelena.pereira@uel.br; 2 Marcos Enrietti Diagnostic Center, Paraná Agricultural Defense Agency, Curitiba 80040-340, Paraná, Brazil; mariaconstanza@adapar.pr.gov.br; 3 Laboratory of Zoonoses and Epidemiology, Department of Preventive Veterinary Medicine, Universidade Estadual de Londrina, Londrina 86057-970, Paraná, Brazil; fpferreira@uel.br; 4 Laboratory of Animal Virology, Department of Preventive Veterinary Medicine, Universidade Estadual de Londrina, Londrina 86057-970, Paraná, Brazil; alaisagnol@uel.br (A.M.D.A.); geovana.depieri@uel.br (G.D.Y.); alfieri@uel.br (A.A.A.); 5 Department of Veterinary Medicine, Universidade Federal do Paraná, Curitiba 80035-050, Paraná, Brazil; renatosousa@ufpr.br

**Keywords:** cervid pathology, free-ranging wild boars, *Macavirus*, molecular characterization, ovine gammaherpesvirus 2, spillover effect, wildlife–livestock interface

## Abstract

Sheep-associated malignant catarrhal fever (SA-MCF) is a frequently fatal, pantropic, lymphoproliferative disease caused by ovine gammaherpesvirus 2 (OvGHV2), with sheep serving as the asymptomatic reservoir host. Although the Rusa deer (*Cervus timorensis*) are known to be highly susceptible to OvGHV2 in other regions, cases have not been documented in this species from Brazil. This report describes the spontaneous occurrence of OvGHV2 infection in a captive Rusa deer from Southern Brazil and discusses possible impacts on wildlife. Three deer from the same property died suddenly without prior clinical signs. These animals had no known contact with sheep or goats, but were maintained in an epidemiological niche where OvGHV2 was detected in subclinically infected free-ranging wild boars (FRWBs) and cattle. One of these was submitted for routine *post-mortem* evaluations. Gross lesions included ulcerative and erosive lesions of the alimentary tract, pulmonary edema, and marked meningeal congestion. Histopathology revealed erosive and ulcerative esophagitis, abomasitis, omasitis, and rumenitis, with nonsuppurative meningoencephalitis, and vasculitis in several organs, consistent with SA-MCF. Molecular testing of brain tissue detected OvGHV2 DNA by qPCR. Furthermore, PCR assays amplified the partial fragments of the OvGHV2 tegument protein (ORF75) and the glycoprotein (gB) genes. Other neurological pathogens of ruminants including BoAHV5, BVDV, *Listeria monocytogenes*, *Histophilus somni*, and rabies virus were not detected. Additionally, phylogenetic analyses, based on ORF75 and gB genes, revealed that the nucleotide sequences derived from this study clustered with and had elevated sequence homology with the OvGHV2 reference strains and other strains derived from ruminants maintained in different geographical locations. The absence of contact with sheep, combined with prior evidence of subclinical OvGHV2 infection in FRWBs and cattle within the same epidemiological niche, suggests the involvement of alternative wildlife reservoirs or bridge hosts in viral transmission. Additionally, these results expand the host range of OvGHV2 in Brazil, highlight the vulnerability of exotic cervids to SA-MCF, and underscore the need for integrated wildlife–livestock surveillance strategies in regions where multiple susceptible species coexist.

## 1. Introduction

Malignant catarrhal fever (MCF) is a severe, frequently fatal, lymphoproliferative infectious disease caused by members of the MCF virus (MCFV) complex of organisms [1,2,3,4]. MCFVs are of epidemiological and economical importance, and are known to cause MCF in mammalian hosts, including ovine gammaherpesvirus 2 (*Macavirus ovinegamma* 2, OvGHV2) and alcelaphine gammaherpesvirus 1 and 2 (*Macavirus alcelaphinegamma* 1 and 2; AlGHV1 and 2), of the *Macavirus* genus, subfamily *Gammaherpesvirinae*, family *Herpesviridae* [5]. Although these MCFVs are known to be associated with the development of MCF in distinct geographical regions, only OvGHV2 has been diagnosed in mammals from Brazil [2]. OvGHV2 produces sheep-associated MCF (SA-MCF), where asymptomatic sheep are the carriers of the virus, with infections occurring in a wide range of dead-end hosts [1,2,3,4], particularly due to contact with the nasal secretions of lambs [6,7]. This theory limits the dissemination of OvGHV2 to a specific group of animals, but does not explain the detection of this pathogen in animals from Brazil [8,9,10,11,12,13] and other geographical locations [14,15,16,17,18] in which there has been no reported presence of the asymptomatic host. Additionally, OvGHV2 is a pantropic virus since lesions associated with concomitant viral detection can occur in most organs and/or tissues [19].

Infections due to OvGHV2 have been diagnosed in a wide range of mammalian species worldwide, including cattle [10,20,21,22], bison [15,23], buffaloes [24,25,26], deer [27,28,29,30,31,32], pigs [33,34,35], and horses [36], with typical clinical manifestations of SA-MCF. Alternatively, subclinical OvGHV2-associated infections have been described in cattle [9,16,37,38,39], buffaloes [16,40], deer [41], free-ranging wild boars (FRWBs) [12,42], and bison [43] from diverse geographical locations. These reports demonstrate the complexity and the difficulty in the diagnosis of infections due to OvGHV2 in animals worldwide, particularly in mammalians without typical clinical manifestations of SA-MCF. In Brazil, outbreaks of SA-MCF and/or subclinical infections associated with OvGHV2 have been diagnosed predominantly in domestic animals, particularly cattle [2], with isolated descriptions in horses [8], buffaloes [24], pigs [44], FRWBs [12,42], and sheep [45,46]. The detection of OvGHV2-related infections in FRWBs from Southern Brazil [12,42] suggests the need to investigate the possible role of these animals in the dissemination of this pathogen [19,47] and the potential spillover effects to wildlife and other domesticated ruminant populations. This is of major importance considering the documented participation of FRWBs in the dissemination of a wide range of bacterial, viral, and protozoan diseases to susceptible animals globally [48,49].

Infections due to OvGHV2 and/or outbreaks of SA-MCF have been described in a wide range of cervids worldwide, including the Rusa deer (*Cervus timorensis*) from Australia [17,27,28], as well as in several types of deer from North America [36,41,50,51,52,53,54,55], Europe [31,56,57], and New Zealand [58]. All types of deer seem to be highly susceptible to MCF [59]. The Rusa deer, also referred to as the Javan deer, Javan rusa, Rusa, Sunda sambar, and Timor deer, is a native *Cervidae* of Indonesia that was introduced to Brazil [60] between 1930 and 1970. Therefore, the Rusa deer are exotic to this country. Additionally, no record of published data relative to the occurrence of diseases in this animal species within Brazil was located when major English and Latin databases were searched.

Two descriptions of OvGHV2-associated infections in deer from Brazil were identified when major English and Latin databases were searched. The first was due to an outbreak in 1994, during which 60% (6/10) of brown brocket deer (*Mazama gouazoubira*) maintained in a petting zoo within the state of Mato Grosso, Midwestern Brazil, died suddenly without any apparent clinical manifestation [61]. The second occurred at a conservationist unit within the state of Rio de Janeiro, Southeastern Brazil, in 2017 where 82.6% (19/23) of sambar deer (*Rusa unicolor*) died after presenting neurological manifestations [62]. In both outbreaks of SA-MCF in deer from Brazil the affected animals were maintained in areas containing sheep [61,62]. Comingling of deer with sheep and/or goats or the maintenance of deer within proximity to sheep and/or goats was the predominant epidemiological feature in spontaneous cases of SA-MCF outbreaks in deer from Australia [17,27], New Zealand [58], North America [50,51,52,53,54,55], and Europe [56,57]. Alternatively, there are reports of SA-MCF in deer without any known contact with sheep and/or goats [17,18,29], suggesting the participation of another mammal host in the dissemination of the disease [18] or alternative forms of transmission [29] in these cases. The objectives of this study were (a) to describe the first documented case of SA-MCF in a captive Rusa deer from Brazil, and (b) to discuss possible implications for wildlife within this specific epidemiological niche.

## 2. Materials and Methods

### 2.1. Study Location, Animals, and Epidemiological Background

During the winter of 2024, three Rusa deer maintained on a private rural property within the municipality of Piraquara died within a period of six weeks. All deer died suddenly without any apparent clinical manifestation of disease. The first animal died in early June, the second in early July, and the third two weeks later. Piraquara is located about 29 km from Curitiba, forms part of the Curitiba metropolitan region in Paraná, Southern Brazil, and is within the Atlantic Forest biome of Brazil. During the winter of 2024, Piraquara had cold mornings and nights followed by mild afternoons, with temperatures varying between 11 and 20 °C, elevated relative humidity (78–84%), and reduced precipitation.

**Figure 1:**
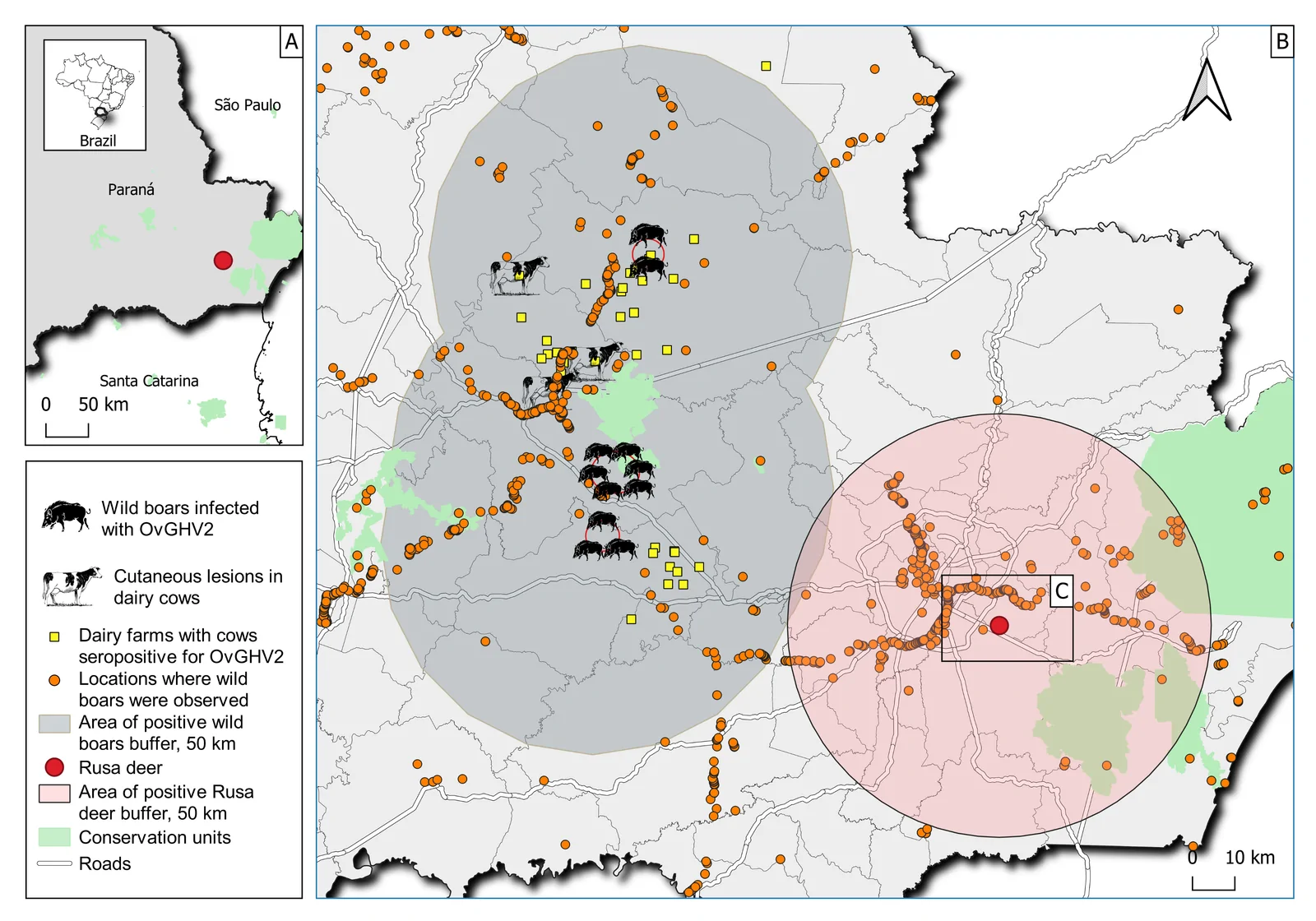
Graphical demonstration of the geographical region where the Rusa deer was maintained. Observe the geographical location of the rural property within the state of Paraná (**A**). The complex epidemiological niche in southern Brazil that contained free-ranging wild boars subclinically infected with OvGHV2, dairy cows with OvGHV2-associated folliculitis, and dairy farms with cows seropositive for OvGHV2 antibodies is shown in (**B**). The location of the property where the Rusa deer was maintained is shown by (**C**). Areas where free-ranging wild boars were reported by Kmetiuk et al. 2023 [49] are included in (**B**,**C**).

Neither sheep nor goats were reared at this property. Previously published data have shown that there was activity of FRWBs (*Sus scrofa*) within the proximity of the rural property where these cervids were maintained [49]. Furthermore, we have demonstrated that FRWBs within this geographical region were subclinically infected by OvGHV2 [12], and that these animals may serve as potential bridge hosts in the dissemination of OvGHV2 to susceptible ruminant populations in areas where sheep are not reared [47]. Additionally, dairy cows without direct contact with sheep and/or goats, that were reared within enclosed farms from this epidemiological niche, developed chronic OvGHV2-related folliculitis [13]. In addition, dairy cows from this geographic area, without contact with sheep, were seropositive to OvGHV2 antibodies [47]. It remains unknown if these cervids were immunized against bovine rabies and other common infectious diseases of ruminants. The geographical location of the rural area within the outskirts of Piraquara is provided in Figure 1. The second deer that died was submitted to the Laboratory of Veterinary Pathology, Universidade Federal do Paraná, Curitiba, for routine pathological evaluations.

### 2.2. Post-Mortem Evaluation and Sampling

An adult female deer was received for *post-mortem* evaluation soon after death. Selected organs were routinely processed for histopathological evaluation via hematoxylin and eosin staining. Fragments of the brain were collected and submitted to the Official Regional State Veterinary Diagnostic Laboratory, Marcos Enrietti Diagnostic Center (CDME-ADAPAR), for the identification of specific infectious disease pathogens of ruminants.

### 2.3. Molecular Detection of Neurological Disease Agents of Ruminants

The nucleic acids from brain fragments were extracted as described in [63], and then used in molecular assays designed to identify specific agents of neurological diseases of ruminants. These included qPCR for OvGHV2 [64], bovine alphaherpesvirus 5, BoAHV5 [65], bovine viral diarrhea virus, BVDV [66], *Listeria monocytogenes* [67], and *Histophilus somni* [68]. A threshold cycle (*Cq)* value ≤ 37 was considered positive for these qPCR assays [69,70].

In addition, PCR analyses were performed to amplify partial fragments of the OvGHV2 tegument protein gene, ORF75 [71], and glycoprotein B (gB) genes. Nucleic acids derived from previous studies [69,70,72] served as positive controls. All PCR products were separated by electrophoresis in 2% agarose gels, stained with ethidium bromide, and examined under ultraviolet light. The negative control for the PCR assay consisted of ultrapure water. Positive and negative controls were included in each molecular assay.

Additionally, brain fragments were processed for the detection of rabies virus by RT-PCR and immunofluorescence assay.

### 2.4. Molecular Characterization of OvGHV2 Tegument Protein and Glycoprotein Genes

The products derived from all molecular assays were purified using the PureLink^®^ Quick Gel Extraction and PCR Purification Combo Kit (Invitrogen^®^ Life Technologies, Carlsbad, CA, USA), quantified by using a Qubit^®^ Fluorometer (Invitrogen^®^ Life Technologies, Eugene, OR, USA), and submitted to direct sequencing in both directions with the forward and reverse primers used in the respective molecular assays in an ABI3500 Genetic Analyzer sequencer with the BigDye Terminator v3.1 Cycle Sequencing Kit (Applied Biosystems^®^, Foster City, CA, USA).

Sequence quality analyses and consensus sequences were obtained using PHRED and CAP3 (http://asparagin.cenargen.embrapa.br/phph accessed on 16 August 2026), respectively. Similarity searches of the OvGHV2 tegument protein gene were performed with nucleotide (nt) sequences deposited in GenBank using BLAST (https://blast.ncbi.nlm.nih.gov/Blast.cgiaccessed on 16 August 2026).

The nt sequences derived from this study were compared with the OvGHV2 reference strains for the tegument protein and glycoprotein genes and strains of OvGHV2 identified in ruminants from Brazil and other geographical locations. The nt sequence identity matrices were constructed using the BioEdit software, version 7.0.8.0. The phylogenetic trees were then generated via the maximum likelihood method based on the Kimura two-parameter model, with 1000 bootstrapped datasets. Phylogenetic analysis of ORF75 was based on 327 nt fragments; the gB analysis consisted of 2164 nt fragments. All evolutionary analyses were conducted with the MEGA10 software.

### 2.5. Spatial Imaging Analyses

The thematic maps were created using QGIS software version 3.40, utilizing the cartographic grid of the Brazilian Institute of Geography and Statistics (IBGE) as a territorial base. Spatial data were organized in a Geographic Information System (GIS) environment using the SIRGAS 2000/UTM zone 22S coordinate reference system (EPSG:31982). The inputs for free-ranging wild boar (FRWB) sightings were obtained from a previous study [49]. Spatial proximity between the deer enclosure, sheep farms, and FRWB sighting areas was assessed through buffer analysis in QGIS, allowing estimation of minimum interaction distances and potential epidemiological overlap between susceptible hosts and possible transmission sources.

## 3. Results

### 3.1. Gross and Histopathological Findings

**Figure 2:**
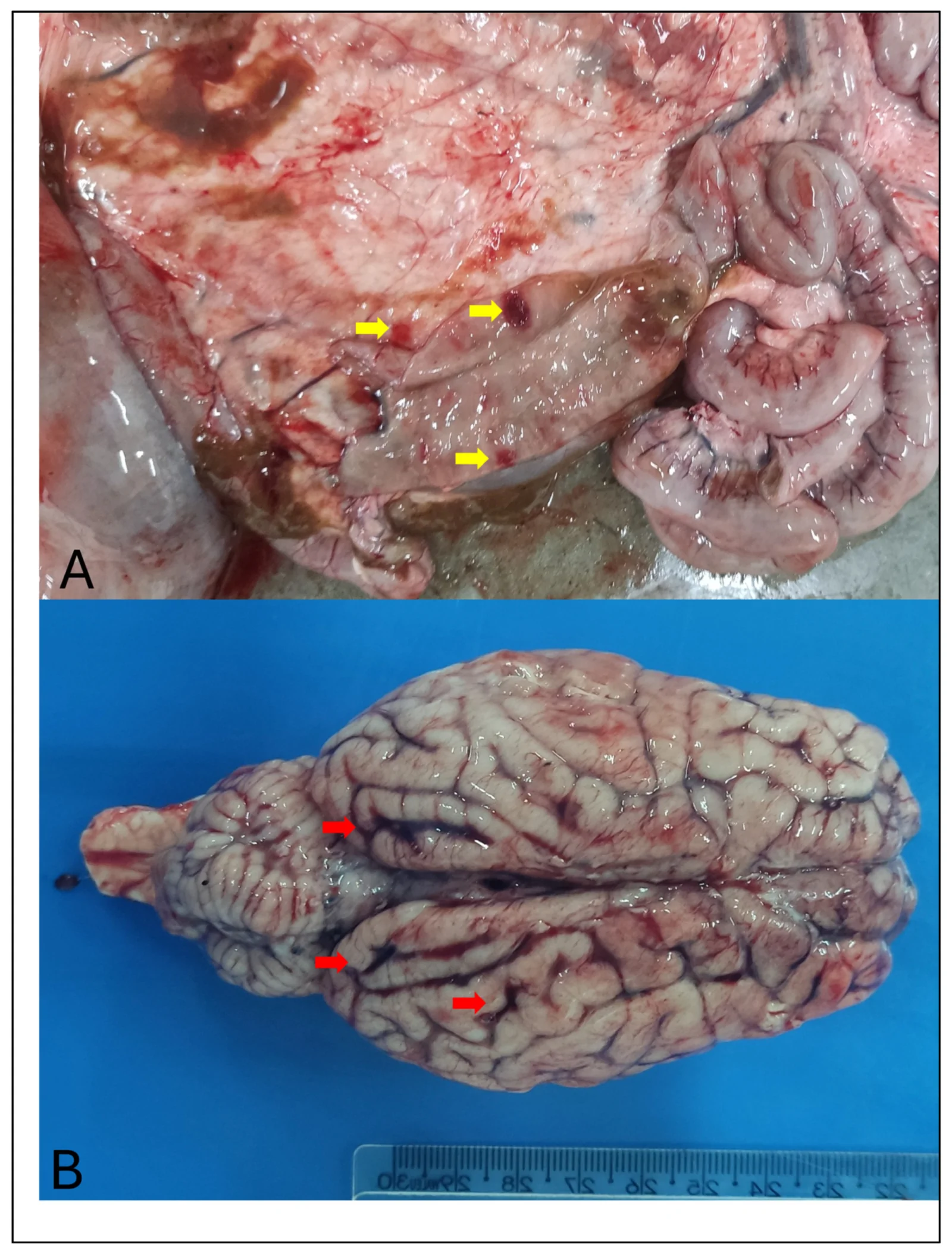
Gross findings observed in a Rusa deer infected with OvGHV2. Observe several ulcers (yellow arrows) at the abomasum (**A**) and marked congestion of the meningeal vessels (red arrows) at the cerebrum (**B**).

The principal gross alterations observed during routine *post-mortem* evaluations of the deer included ulcerative and/or erosive lesions at the esophagus, rumen, abomasum (Figure 2A), reticulum, and omasum, with pulmonary edema and congestion. The vessels of the meninges were severely congested and there was marked distension of the cerebral gyri and shallow sulci (Figure 2B). Additionally, the liver was slightly enlarged with several pale areas at the sectioned surface.

**Figure 3:**
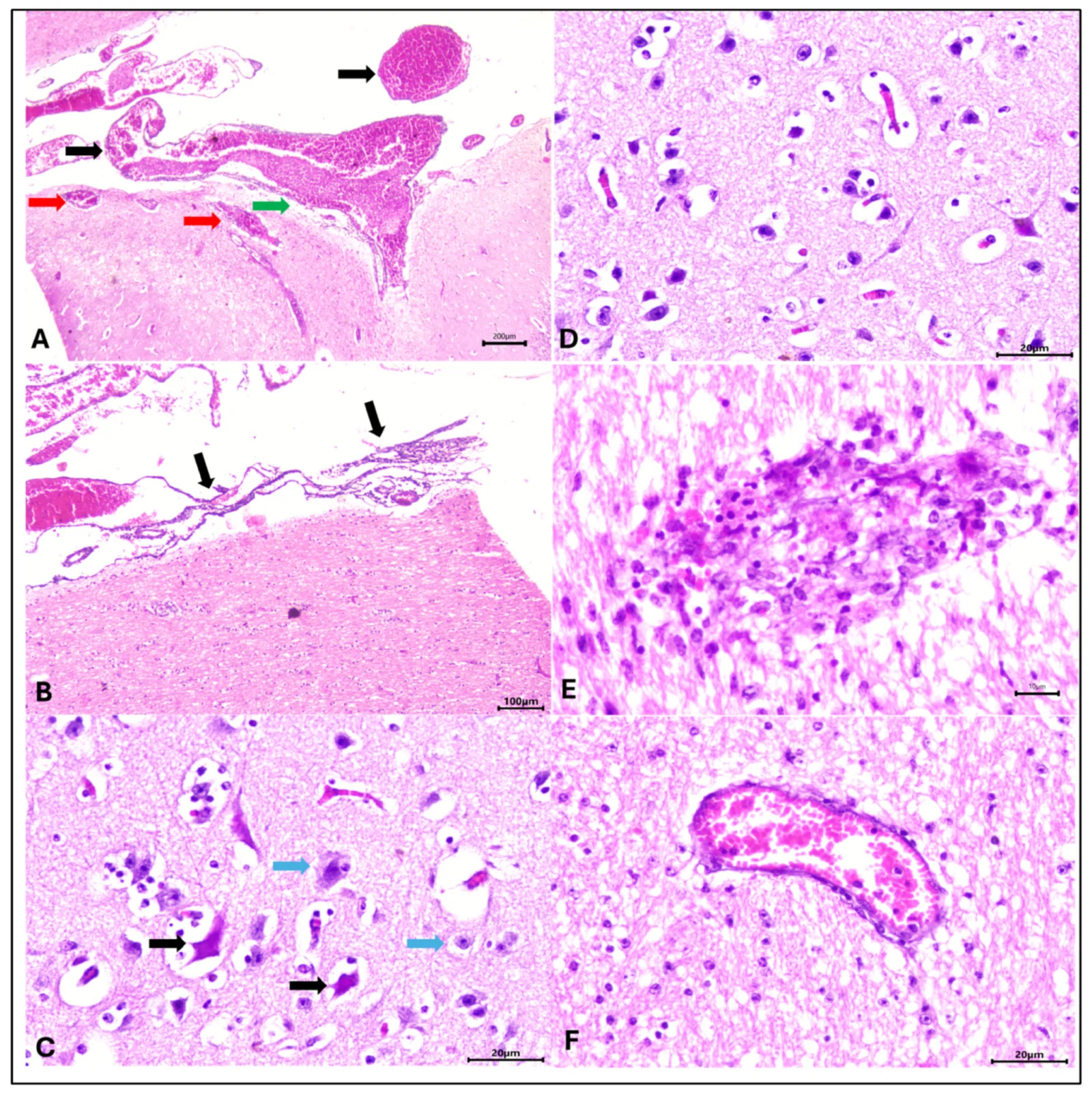
Histopathological findings observed in the cerebrum of a Rusa deer infected with OvGHV2. (**A**) Congestion of vessels at the meninges (black arrows) and the neuropil (red arrows), and lymphocytic meningitis (green arrow). Meningitis (arrows) is more easily appreciated (**B**). Observe neuronal necrosis (black arrows) compared with normal (bluarrows) neurons (**C**). There is brain edema (**D**), vasculitis (**E**), and a perivascular cuff with a few lymphocytes. Hematoxylin and eosin stain. Bars: (**A**) 200 µm; (**B**) 100 µm; (**C**,**D**,**F**) 20 µm; (**E**) 10 µm.

Histopathology revealed erosive and ulcerative esophagitis, abomasitis, omasitis, and rumenitis; moderate portal lymphoplasmacytic hepatitis; and mild, multifocal, lymphoplasmacytic interstitial nephritis. The histopathological changes observed in the brain (cerebrum and cerebellum) were more severe in the cerebrum (Figure 3). These encephalitic alterations consisted of several perivascular cuffings formed by one layer of lymphocytes, foci of neuronal necrosis, areas of moderate cortical edema, moderate-to-severe congestion of the neuropil and meninges, and mild lymphoplasmacytic infiltration of the meninges, resulting in mild-to-moderate, multifocal, lymphoplasmacytic meningoencephalitis. Additional significant lesions included lymphoplasmacytic vasculitis in the lungs.

### 3.2. Molecular Detection of OvGHV2

The OvGHV2 qPCR assay detected a *Cq* of 26.61 from the brain fragments of the deer, demonstrating that an elevated number of viral particles was detected by this molecular assay. The PCR assays amplified the desired partial nt of ORF75 and the gB gene. The ORF75 and gB strains identified in this study were named OvGHV2/BRA/PR-Rusa-Deer/24. These sequences were deposited in GenBank (ORF75, PZ896339; gB, PZ878946).

Genetic material was not amplified by the *H. somni*, *L. monocytogenes*, BVDV, and BoAHV5 qPCR assays. Additionally, rabies virus was not identified by the immunofluorescence assay or by RT-qPCR at the Official Regional State Veterinary Diagnostic Laboratory.

### 3.3. Molecular Characterization of Ovine Gammaherpesvirus 2 ORF75 and gB Strain

**Figure 4:**
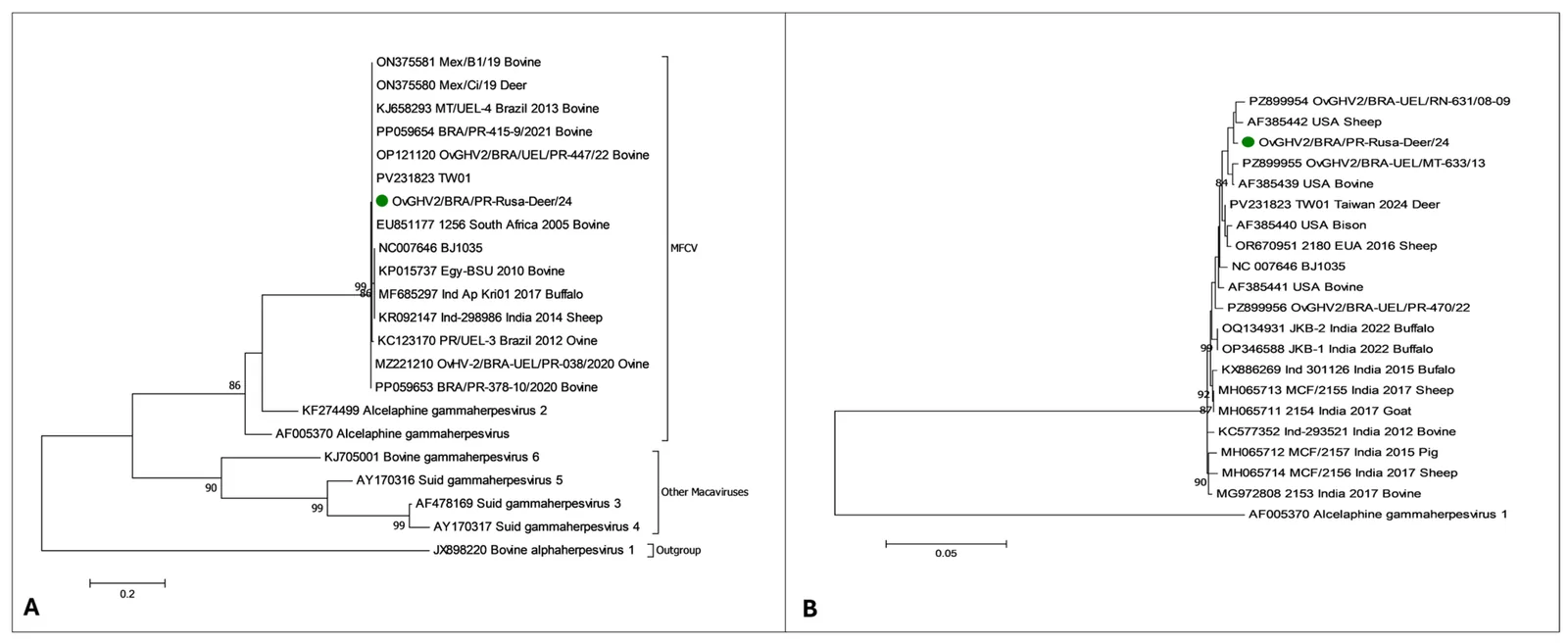
Phylogenetic analyses of the ovine gammaherpesvirus 2 based on the tegument protein (**A**) and glycoprotein B (**B**) genes using selected strains derived from diverse geographical locations. The strains identified in this investigation are identified (●). Bovine alphaherpesvirus 1 served as the outgroup for the ORF75 analysis; alcelaphine gammaherpesvirus 1 was used as the outgroup for the glycoprotein B analysis.

Phylogenetic analysis based on ORF75 revealed that three somewhat distinct clusters formed by the MCFV derived from diverse geographical locations (Figure 4A). The strain herein identified clustered with OvGHV2 strains identified in a deer (ON375580) and cow (ON375581) from Mexico; a Formosan sambar deer from Taiwan (PV231823); a cow from South Africa (EU851177); and cows from the Atlantic (OP121120; PP059654) and Amazon (KJ658293) biomes of Brazil. The second cluster contained the OvGHV2 reference strain (NC007646) and strains identified in ruminants from diverse geographical locations. The third cluster consisted of OvGHV2 strains detected in cattle (PP059653) and sheep (MZ221210) from the Atlantic Forest biome of Brazil.

Furthermore, analysis of the nt sequence identity table (not provided) of the ORF75 gene sequences revealed that this strain had 100% nt sequence identity with the deer (ON375580) and cow (ON375581) from Mexico, the Sambar deer (PV231823) from Taiwan, the cow from South Africa, as well as cows from the Atlantic (OP121120; PP059654) and Amazon (KJ658293) biomes of Brazil. In addition, the strain herein identified had 99.3% nt sequence similarity with the OvGHV2 reference strain (NC007646).

The gB phylogenetic analysis (Figure 4B) revealed that the strain derived from this study formed a subcluster that contained strains identified in cattle from the Amazonia (UEL/MT) and Caatinga (UEL/RN) biomes of Brazil, as well as in sheep (AF385442) and cattle (AF385439) from the USA. This subcluster seems to have emerged from a cluster containing the OvGHV2 reference strain (NC007646), and gB strains identified in a Formosan sambar deer (PV231823) from Taiwan, and with cattle (AF385441), sheep (OR670951), and bison (AF385440) from the USA. In addition, there was a somewhat distinct cluster formed by a wide range of animals (sheep, buffalo, pig, and goat) from India.

Analysis of the gB nt sequence identity (not provided) revealed that the strain herein identified had 99.4% homology with strains derived from the Caatinga biome (UEL/RN) of Brazil and sheep (AF385441) from the USA, and 99.3% nt sequence similarity with strains identified in sheep (OR670951) and cattle (AF385439) from the USA, as well as the deer (PV231823) from Tiwan. Moreover, the OvGHV2/BRA/PR-Rusa-Deer/24 strain had 99.2% nt sequence identity with strains from the bison (AF385440) and cattle from the Amazon biome (UEL/MT), and 98.8 nt homology with the OvGHV2 gB reference strain (NC007646).

### 3.4. Epidemiological and Spatial Imagining Findings

**Figure 5:**
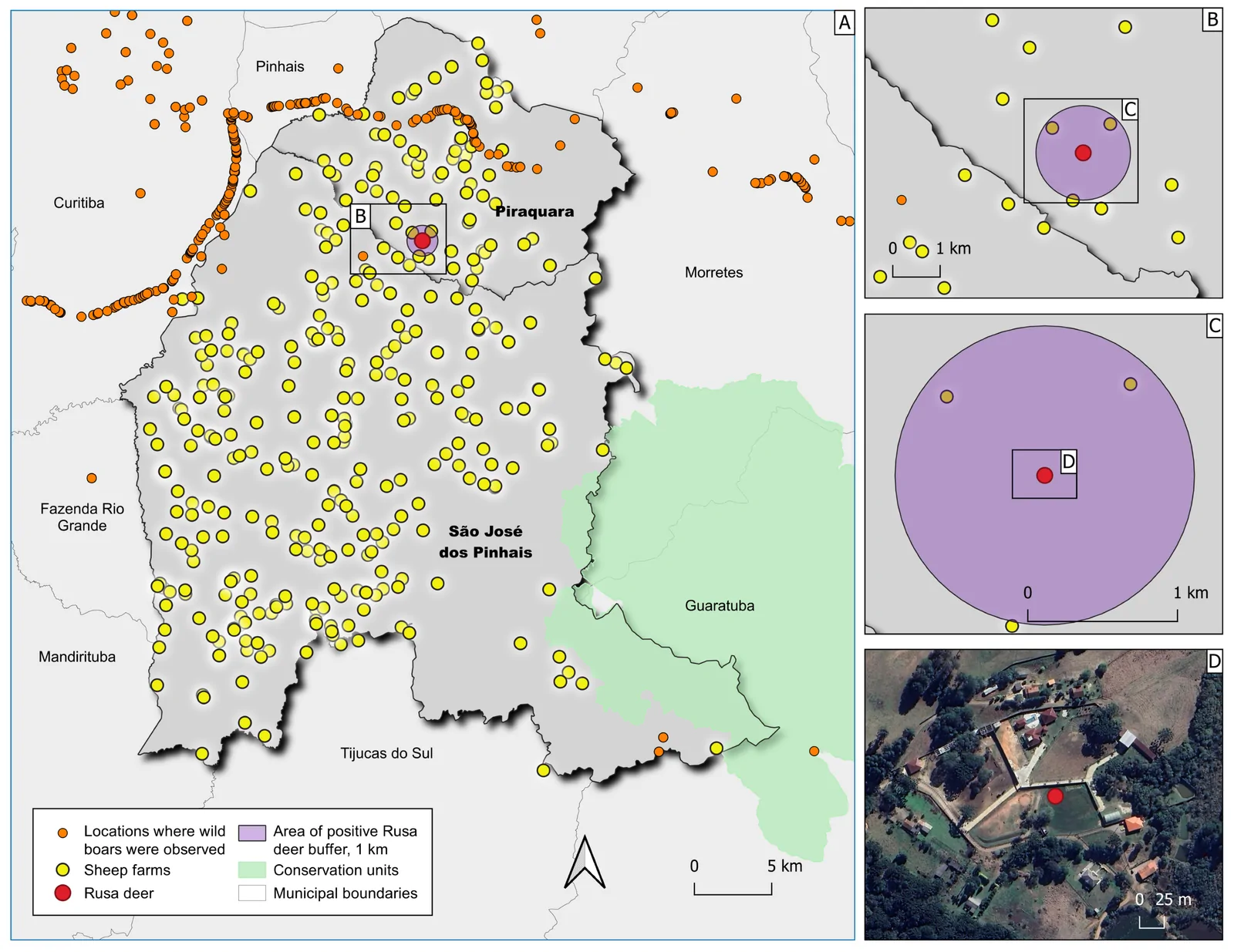
Geographical representation of the study area within Paraná state, Southern Brazil, where SA-MCF was detected in a captive *Rusa deer*. Spatial distribution of sheep farms and sightings of free-ranging wild boars (**A**) within the epidemiological niche. Closer spatial views where the *Rusa deer* was maintained and the relationship between the sheep farms and areas where free-ranging wild boars were sighted (**B**,**C**). Aerial image of the property (**D**).

Agriculture census data received from ADAPAR revealed that this geographical niche contained numerous farms and/or rural properties where sheep were reared (Figure 3A,B) and areas where FRWBs were sighted [49]. Sheep rearing within this region is based on a semi-intensive system in which animals are maintained within enclosed areas, most of these by some form of fencing. Spatial analysis revealed that FRWBs were observed less than 5 km distant from the habitat of the Rusa deer (Figure 5A), while the closest sheep farm was at least 1 km distant from the Rusa deer (Figure 5B,C).

Additionally, the cervids at this property were maintained in an enclosed area that was partially surrounded by a concrete fence on the southern part of the property and wire-based fencing on the northern side (Figure 5D). In addition, native and planted forests were present near the property.

## 4. Discussion

The present report documents the spontaneous occurrence of OvGHV2 infection in a captive Rusa deer from Southern Brazil. These findings represent the first description of SA-MCF in this species within the country, the second confirmation of OvGHV2-induced infection worldwide in this type of deer, and the second detection of SA-MCF in a Rusa deer distant from its natural habitat. These findings are epidemiologically significant since the affected deer had no known contact with sheep or goats, and are contrary to the results of the two previous descriptions of SA-MCF in deer from Brazil [61,62] as well as the Rusa deer from Australia [27], in which the infected deer had contact with sheep. However, the epidemiological context of this case suggests that the classic sheep–susceptible hosts interface does not fully explain viral exposure. Accordingly, these findings challenge the traditional understanding of SA-MCF transmission dynamics, and reinforce the need to consider alternative epidemiological pathways, prompting the need to consider additional hosts capable of maintaining or disseminating OvGHV2 in this region.

The gross and histopathological findings observed in this deer are consistent with those described in ruminants infected with OvGHV2 [1,2,3]. The amplification of OvGHV2 DNA by several molecular assays confirmed that this animal was infected by this pathogen; similar reports have been described in a wide range of mammals worldwide [15,35,36,53,56]. The utilization of pathological findings combined with molecular testing is recommended for the confirmation of a diagnosis of OvGHV2-induced infection and/or SA-MCF [2,3,4,73]. Additionally, phylogenetic investigations with two distinct genes demonstrated that the OvGHV2 strain herein identified clustered with and had elevated nucleotide similarity with the reference strain and other strains derived from distinct geographical locations; similar findings have been described [36,72,74,75]. In addition, some clusters contained a mixture of mammalian species, including sheep, cattle, and bison; similar findings have been reported in studies conducted in India [74,75]. The clustering of multiple species by the gB gene analysis, as herein detected, was related to host adaptation and possible cross-species transmission [74]. Moreover, phylogenetic analysis with the gB in cattle from India has identified specific amino acid substitutions which suggested that infection occurred by distinct OvGHV2 variants [75]. However, detailed analyses of the gB sequence herein identified were not within the scope of the current investigation. Nevertheless, we are currently analyzing the complete gB sequences derived from ruminants maintained in the Amazon, Caatinga, and Atlantic Forest biomes of Brazil to detect possible differences from the strains derived from these distinct geographical locations of continental Brazil (article in preparation). This is because the initial analyses herein presented with the gB gene revealed that the strains from these distinct biomes were not within the same cluster and could have evolved differently, even though there are small differences in the homology of the nucleotide and amino acid sequences from the strains identified in these biomes [72]. Nonetheless, evolutionary inferences of OvGHV2 strains require the analysis of specific loci of this organism [76,77]. Additionally, the sequences derived from MCFV-induced infections in deer seem to form a distinct clade from other related viruses [59].

The widespread ulcerative lesions observed in the alimentary tract of this deer were frequently described in animals with the head-and-eye-form of SA-MCF [2,3,36,78]. This may suggest that this animal was affected by the most frequent manifestation of SA-MCF. The histopathological findings herein demonstrated have been reported in deer [30,31,32] and ruminants [2,3] infected with OvGHV2. Additionally, the non-amplification of the nucleic acids of *H. somni*, *L. monocytogenes*, BVDV, and BoAHV5 by their respective molecular assays, as well as the non-detection of rabies virus, suggests that these common pathogens of ruminants were not involved with the development of the pathological findings herein described. Consequently, this report represents one of the few cases to confirm the participation of OvGHV2 in wildlife from Brazil, considering that only two previously published reports have been described in cervids [61,62] from this continental nation. In a wider perspective, this case probably represents the third characterization of OvGHV2 and/or SA-MCF in deer from South America.

The detection of OvGHV2 in a deer reared under enclosed conditions and without any known contact with sheep raises important questions regarding alternative transmission pathways for SA-MCF in Southern Brazil. Accordingly, the exact form of dissemination remains obscure. The non-detection of the source of infection herein described has been reported in previous outbreaks of SA-MCF and/or infections by OvGHV2 in Brazil [8,9,10,11,12,13,19] as well as in other countries [14,15,16,17,18]. This has resulted in the need to identify additional forms of transmission or a possible mammalian host that may participate in the epidemiology of SA-MCF and/or infections due to OvGHV2. In this regard, there is evidence to suggest the possible involvement of FRWBs as potential participants in the epidemiological cycle of OvGHV2 in Southern Brazil [12,19,47].

In this case, the three Rusa deer maintained at this property died suddenly; peracute manifestation is typical of SA-MCF in deer [59]. Considering that the sudden death of these animals occurred within six weeks, it is probable that all animals were infected with OvGHV2. Accordingly, the OvGHV2-associated morbidity could have been 100%, with 100% mortality and lethality. Alternatively, if only the animal that was evaluated was considered, morbidity would have been 33.3%, with 100% mortality and lethality. However, caution must be taken in the interpretation of these indices due to the relatively small number of animals. This spontaneous occurrence of SA-MCF with elevated morbidity, mortality, and lethality is contrary to the typical epidemiological features of SA-MCF in Brazil [2] and other geographical locations [1,3,78]. Although no clinical manifestations were reported in the current case, the inflammatory reactions observed within the brain suggest that the animal suffered an acute neurological syndrome; brain involvement is part of the head-and-eye form of SA-MCF [1,4,78]. Additionally, deer spontaneously infected with OvGHV2 normally succumb without demonstrating the typical clinical manifestations of SA-MCF [27,59], as occurred in the current case.

### Implications for Wildlife Management and One Health in Brazil

The detection of OvGHV2 in a deer maintained under enclosed, sheep-free conditions underscores the need to reassess current wildlife-health surveillance frameworks in Southern Brazil. The emerging evidence that FRWBs may act as alternative hosts or bridge species for OvGHV2 transmission [12,19] has direct implications for the management of both wildlife and livestock populations. Since FRWBs are highly mobile, adaptable, and capable of occupying agricultural, peri-urban, and natural landscapes [49], their potential involvement in the epidemiological cycle of SA-MCF [19] introduces new challenges for disease monitoring and control. Nevertheless, the FRWB theory as possible bridge host for OvGHV2 dissemination in the absence of a known asymptomatic host [12,19,47] warrants additional investigation to be validated. However, it must be highlighted that FRWBs are known to be disseminators of several diseases, including classic swine fever virus, porcine circovirus, influenza A virus, hepatitis E virus [48], toxoplasmosis, salmonellosis, leptospirosis, brucellosis, tuberculosis, and trichinellosis [49]. Therefore, the possibility of these animals serving as potential disseminators of OvGHV2 cannot be totally ignored.

In addition to the FRWB theory [19], other nonconventional methods of OvGHV2 dissemination to susceptible ruminant populations in the absence of sheep were proposed in studies derived from distinct geographical locations. These theories include (a) aerosol dissemination and/or the participation of birds and other fomites during an outbreak of SA-MCF in bison from the USA [14]; (b) direct transmission between a subclinically infected water buffalo, acting as reservoir host, to other buffalos in Switzerland [16]; (c) direct contact between deer in an outbreak of SA-MCF in New Zealand [58]; (d) dissemination between bison resulting in severe bison mortality in the USA [15]; and (e) possible cow-to-cow transmission in dairy cattle from Southern Brazil [47]. Collectively, these investigations suggest that the epidemiology of OvGHV2-induced infection and/or SA-MCF is not well-defined, and that viral transmission may be adapted in specific epidemiological niches. Therefore, additional studies must be performed to fully understand the complex dynamics of the epidemiological features of this disease. Accordingly, sheep and/or goats may not be the only mammalian species that can serve as asymptomatic hosts for OvGHV2.

Notwithstanding the above, this report highlights the complexity of pathogen transmission at the wildlife–livestock interface in Southern Brazil. The confirmation of OvGHV2 in multiple species within the same ecological niche, including dairy cattle with chronic folliculitis [13], seropositive dairy cows without contact with sheep [47], subclinically infected FRWBs [12], and now a Rusa deer, may suggest that the virus may be more widely distributed in Brazil than previously recognized. Accordingly, the detection of this pathogen in additional wildlife species from Brazil, or this specific epidemiological niche, is likely to occur. In the current study, all deer probably died due to SA-MCF. Furthermore, OvGHV2-induced infections have had similar devastating effects on wildlife in the two previous outbreaks of SA-MCF in Rio de Janeiro [62] and Cuiabá [61], resulting in elevated morbidity and lethality (morbidity, 60–83%; lethality, 100%). The elevated morbidity indices described in these deer populations contrasts with the relatively reduced morbidity (1.8–40%) observed in cattle from diverse geographical regions of Brazil [2]. In these outbreaks, the susceptible deer populations were reared with sheep and probably suffered from the spillover effect of interspecies contamination. Likewise, buffalos from Minas Gerais that died due to SA-MCF had reduced mortality (3.5%; 5/145) but 100% lethality, and were also reared concomitantly with the asymptomatic host [24]. Therefore, the spillover effect seems to be responsible for the occurrence of SA-MCF in non-cattle ruminants from Brazil.

From a management perspective, the presence of OvGHV2-positive FRWBs near conservation units, farms, and livestock operations suggests that disease-risk assessments must extend beyond traditional reservoir species. Nevertheless, from a One Health perspective, the findings of the current study underscore the need for the following: (a) enhanced surveillance of OvGHV2 in wildlife and livestock; (b) farm–wildlife interface management, with separation and restriction of contact between sheep and susceptible wildlife populations to prevent spillover; (c) biosecurity measures to limit wildlife access to captive cervid enclosures; (d) monitoring populations of FRWBs, including molecular screening for OvGHV2 and other pathogens of veterinary importance, considering that these animals are expanding and recognized as invasive species; and (e) increased awareness among veterinarians, diagnosticians, and wildlife managers regarding atypical transmission routes.

Finally, these findings highlight the importance of integrated, multi-species health management approaches. Furthermore, collaboration between wildlife agencies, livestock producers, hunters, and environmental authorities will be essential to address the ecological complexity introduced by invasive wild boars.

## 5. Conclusions

This case expands the epidemiological understanding of OvGHV2 in Brazil by demonstrating that SA-MCF can occur in exotic cervids even in the absence of direct contact with sheep, suggesting alternative transmission pathways possibly involving wildlife reservoirs. These findings emphasize the importance of integrated surveillance and management strategies to mitigate the spread of OvGHV2 in regions where wildlife, livestock, and captive exotic species coexist.

Moreover, the detection of OvGHV2 in a deer maintained under enclosed conditions and without any known exposure to sheep challenges the traditional understanding of SA-MCF transmission in Southern Brazil. This case provides evidence that the epidemiological cycle of OvGHV2 in the region extends beyond the classic sheep–susceptible host interface. Therefore, the need to identify alternative forms of dissemination must be thoroughly investigated. Consequently, continued research into the ecology of OvGHV2 across diverse host species will be critical for understanding the full scope of transmission routes, and for developing more effective measures to mitigate the impacts of MCF in this region.

### Study Limitations

This study has one major limitation that should be considered when interpreting the findings. This report describes a single case of OvGHV2-induced infection in a captive Rusa deer, while the two additional animals were not evaluated for possible detection of OvGHV2. In this case, detection of OvGHV2 in the other deer was not possible since the owner did not submit the other animals for pathological evaluation. However, since all deer died suddenly within a six-week period, and deer are highly susceptible to infection by OvGHV2, it is plausible to suggest that all animals were infected by this organism. Nevertheless, these findings expand the host range for OvGHV2 in Brazil and challenge the traditional dissemination pathway, with the FRWB hypothesis.

## Data Availability

The sequences derived from this study were deposited in GenBank.
